# Genome-wide variants and optimal allelic combinations for citric acid in tomato

**DOI:** 10.1093/hr/uhae070

**Published:** 2024-03-18

**Authors:** Wenxian Gai, Liangdan Yuan, Fan Yang, John Kojo Ahiakpa, Fangman Li, Pingfei Ge, Xingyu Zhang, Jinbao Tao, Fei Wang, Yang Yang, Yuyang Zhang

**Affiliations:** National Key Laboratory for Germplasm Innovation & Utilization of Horticultural Crops, Huazhong Agricultural University, Wuhan 430070, China; National Key Laboratory for Germplasm Innovation & Utilization of Horticultural Crops, Huazhong Agricultural University, Wuhan 430070, China; College of Horticulture, Northwest A&F University, Yangling 712100, China; National Key Laboratory for Germplasm Innovation & Utilization of Horticultural Crops, Huazhong Agricultural University, Wuhan 430070, China; National Key Laboratory for Germplasm Innovation & Utilization of Horticultural Crops, Huazhong Agricultural University, Wuhan 430070, China; National Key Laboratory for Germplasm Innovation & Utilization of Horticultural Crops, Huazhong Agricultural University, Wuhan 430070, China; National Key Laboratory for Germplasm Innovation & Utilization of Horticultural Crops, Huazhong Agricultural University, Wuhan 430070, China; National Key Laboratory for Germplasm Innovation & Utilization of Horticultural Crops, Huazhong Agricultural University, Wuhan 430070, China; National Key Laboratory for Germplasm Innovation & Utilization of Horticultural Crops, Huazhong Agricultural University, Wuhan 430070, China; National Key Laboratory for Germplasm Innovation & Utilization of Horticultural Crops, Huazhong Agricultural University, Wuhan 430070, China; National Key Laboratory for Germplasm Innovation & Utilization of Horticultural Crops, Huazhong Agricultural University, Wuhan 430070, China; Hubei Hongshan Laboratory, Wuhan 430070, China; Shenzhen Institute of Nutrition and Health, Huazhong Agricultural University, Wuhan 430070, China; Shenzhen Branch, Guangdong Laboratory for Lingnan Modern Agriculture, Genome Analysis Laboratory of the Ministry of Agriculture, Agricultural Genomics Institute at Shenzhen, Chinese Academy of Agricultural Sciences, Shenzhen 518000, China

## Abstract

Citric acid (CA) plays a crucial role as a fruit flavor enhancer and serves as a mediator in multiple metabolic pathways in tomato fruit development. Understanding factors influencing CA metabolism is essential for enhancing fruit flavor and CA-mediated biological processes. The accumulation of CA, however, is influenced by a complex interplay of genetic and environmental factors, leading to challenges in accurately predicting and regulating its levels. In this study, we conducted a genome-wide association study (GWAS) on CA, employing six landmark models based on genome-wide variations including structural variants, insertions and deletions, and single nucleotide polymorphisms. The identification of 11 high-confidence candidate genes was further facilitated by leveraging linkage disequilibrium and causal variants associated with CA. The transcriptome data from candidate genes were examined, revealing higher correlations between the expression of certain candidate genes and changes in CA metabolism. Three CA-associated genes exerted a positive regulatory effect on CA accumulation, while the remaining genes exhibited negative impacts based on gene cluster and correlation analyses. The CA content of tomatoes is primarily influenced by improvement sweeps with minimal influence from domestication sweeps in the long-term breeding history, as evidenced by population differentiation and variants distribution. The presence of various causal variants within candidate genes is implicated in the heterogeneity of CA content observed among the tomato accessions. This observation suggests a potential correlation between the number of alternative alleles and CA content. This study offers significant function-based markers that can be utilized in marker-assisted breeding, thereby enhancing their value and applicability.

## Introduction

The acidity levels in mature tomato fruits play a pivotal role in discerning the flavor profile and their nutritional composition, which are predominantly malic and citric acids (organic acids) [1, 2]. Citric acid (CA) is dominant in many fruits and contributes to fruit acidity [3, 4]. CA has a marked influence on consumers’ preferences [5]. The sensation induced by CA demonstrates a prompt initiation momentarily, concurrently modulating the thresholds for perceiving sweetness, sourness, astringency, and bitterness [6]. The CA content in fruits has a direct impact on their quality. A high concentration of CA can effectively inhibit the dehydration and granulation of oranges, prevent browning in fruits and vegetables, and enhance overall taste and flavor [7]. CA also serves as a mediator of key metabolism pathways in the mesocarp cells of fleshy fruits, including tricarboxylic acid (TCA) and glyoxylate cycles [2, 3]. Therefore, understanding the factors influencing CA metabolism is crucial for enhancing fruit flavor and its role in tomato fruit quality.

The content of CA in fruit is determined by a complex and stable enzymatic regulatory system that controls its synthesis, degradation, and transport [3, 8]. The conversion of CA from malate and oxaloacetate can occur via two metabolic pathways, namely the TCA and glyoxylate cycles, facilitated by NAD-malate dehydrogenase and citrate synthase (CS) [9, 10]. Again, citrate can be converted into dicarboxylates via acetyl-CoA catabolism, TCA and glyoxylate cycles, and gamma-aminobutyric acid pathway [3, 8]. In this process, the enzyme aconitase (ACO) directly facilitates the reversible conversion of citrate to isocitrate. Mitochondrial ACO is involved in the TCA cycle [11], while cytosolic ACOs function via the gamma-aminobutyric shunt [12]. The majority of CA content in fruit is predominantly located within the vacuole, and the transport of CA into the vacuole appears to occur readily once its cytosolic concentration reaches a sufficient level [13]. The transport rate of CA and its precursors within fruit cells determines the extent of CA accumulation in the vacuole, as their synthesis and metabolism necessitate movement between different cellular compartments. For example, the *Arabidopsis* tonoplast dicarboxylate transporter *AttDT* is involved in the transport of citrate into the vacuole [14], while overexpression of the *SlTDT* gene significantly reduces CA content in tomato fruit [15]. The citrate transporter protein and the dicarboxylate-tricarboxylate carrier also play a crucial role in mediating the transport of CA within mitochondria [3]. The concentration of CA in fruit cells can be directly altered by these influencing factors. Transcription factors play an important role in the regulation of CA concentration in fruit. The synergistic action of *Cit*NAC6 and *Cit*WRKY1 was found to modulate the accumulation of CA degradation by up-regulating the expression of *CitAco3* [16]. ACO promoters are also regulated for CA accumulation by bHLH35, NAC7, HLH113, and TRY transcription factors through their interactions [17]. In tomato, the suppression of the MADS-Box transcription factor gene led to an up-regulation of CA degradation-related gene [18]. Additionally, certain candidate genes have also been proposed to govern the accumulation of CA. The overexpression of *PpTST1*, a tonoplast sugar transporter in peach, results in a decrease in CA content in both peach and tomato [19], and the P-type proton pump gene, *CsPH8*, plays a pivotal role in the differential accumulation of CA in citrus fruits [20].

The metabolic accumulation of CA in fruits is regulated by both genetic and environmental factors [3, 21], with an estimated heritability of 0.54 [22]. The estimated broad-sense heritability for the content of malic acid ranged from 0.19 to 0.66 [4, 23, 24]. Although the mechanism of CA metabolism is relatively well understood, there exists a significant research gap regarding its genetic underpinnings. GWAS offers significant insights into comprehending the genomic-level natural variation and genetic development of CA metabolism in tomatoes [4, 22, 24, 25]. In certain studies, no variation sites were identified to be associated with CA content [24], or the yield of significant variant sites associated with CA content limited to only one [25]. The statistical power of GWAS is impeded by several factors, including incomplete detection of genomic variants, selection bias in association models, phenotypic values in specific environments, as well as genetic heterogeneity among causal variants. The environmental factors exert a substantial influence on the contribution to variance of associated loci for quantitative traits [23]. The heritability of molecular traits in GWAS analyses is influenced by variants in single-nucleotide polymorphisms (SNPs), insertions and deletions (Indels), as well as structural variants (SVs) [26]. However, GWAS were primarily conducted utilizing SNPs to identify significant associations in the majority of prior surveys [4, 5, 23, 24]. The significant SNP variants associated with tomato CA concentration have been identified using single models mixed linear model (MLM) [5,22] and multiple loci mixed model (MLMM) [24, 25]. The number of identified significant association loci, however, varied significantly across studies, ranging from no associated SNP to several dozen associated SNPs, indicating a genetically diverse and complex foundation underlying tomato CA content.

The primary objective of this study was to identify the associated loci with CA content in mature tomato fruits and elucidate the polygenic architecture governing its regulation. We conducted a comprehensive identification of genetic variants (SNPs, Indels, and SVs) across the entire genome and performed a GWAS on CA using six landmark-mapping models. The GWAS analysis revealed a series of associated natural variants, and subsequently, high-confidence annotated genes were identified based on the potentially causal variants. The genetic parameters of population differentiation were utilized to identify potential signals of selective sweeps on CA during the processes of domestication and improvement. An assessment of superior alleles that regulate the accumulation of CA was undertaken for their potential value in tomato breeding. Our study offers novel genetic insights and identifies potential functional genes associated with tomato CA, thereby presenting a valuable resource for enhancing tomato crop improvement.

## Results

### GWAS detection of multiple loci underlying tomato citric acid

**Figure 1:**
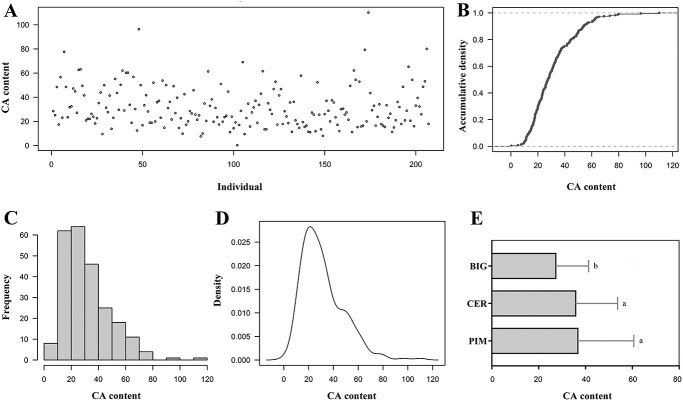
The phenotype of CA content in GWAS population. The accumulative density (**B**), frequency distribution (**C**), and density (**D**) of the CA content among tomato individuals (**A**). (**E**) Distributions of CA content among different tomato accessions. Different lowercase letters indicate significant differences at *P* ≤ 0.05 by *t* test.

The present study revealed a wide phenotypic range for CA content in tomato germplasm resources (Fig. 1A and B), indicating significant genetic variants in the genetic loci controlling CA content among these accessions. Heritability estimate for CA content was 0.35 with a standard error of 0.11. The individual heterozygosity of the tomato accessions did not exceed 0.6, with most germplasms exhibiting a heterozygosity ranging from 0 to 0.1 (Fig. S1A, see online supplementary material). The phenotypic values exhibited a normal distribution pattern in their frequency distribution (Fig. 1C and D). The distributions of CA content within various tomato accessions were evaluated (Fig. 1E). The CA content exhibited no significant difference between PIM and CER, whereas the content in BIG accessions was statistically lower compared to that in PIM and CER. The phenotypic data were utilized to dissect complex traits through association analyses, taking into account the population structure and size within each phenotypic class. Subsequently, all tomato accessions were genotyped. After applying a filtering process, we obtained a high-density genotyping subset consisting of 4 353 430 variants, which included 4 063 982 SNPs, 234 331 Indels, and 55 117 SVs (Table S1, see online supplementary material). The distribution of genome-wide variations across 12 chromosomes is illustrated in Fig. S2 (see online supplementary material). The distribution of most of the variants minor allele frequency (MAF) ranges from 0.05 to 0.15 (Fig. S1B, see online supplementary material).

The GWAS analysis involved the utilization of six distinct association mapping models to investigate the association between phenotypes and genotypes. About 26 significant variants (*P* < 4.52 × 10^−7^) were identified from the GWAS results (Table S2, see online supplementary material). While all six models were based on the same thresholds (Table 1; Table S2 and S3, see online supplementary material), they respectively discovered different sets of suggestive variants linked with CA content, which may be due to the differing statistical power of the GWAS models. More than one suggestive linked variant was identified with any model. Especially, the single-locus models detected a greater number of variants compared to the multiple-locus models, surpassing the suggestive threshold of -Log10 (*P*) > 6.34. The point worth highlighting is that the single-locus models failed to identify any sites, when a significant threshold of -Log10 (*P*) > 7.65 was applied. The number of significant loci identified by single loci models was obviously more than that by multiple-loci models (Table S2, see online supplementary material). The single-locus model, MLM and GLM (general linear model) yielded a greater number of variants due to the fact that one variant within these peaks exhibited the highest correlation with CA content, while the other variations within the given peak were in strong linkage disequilibrium (LD) with the peak variants (Table S4, see online supplementary material). These findings suggest that the diversity of GWAS results significantly varies depending on the employed types of association mapping models, emphasizing the importance of the inherent characteristics of the model itself when selecting candidate genes.

**Table 1:** Variants associated with tomato CA

Types^a^	Loci	Lead variants	Causal variants				Model	-log10 (*P*)	Candidate genes^b^
			ID	Ref	Alt	Location			
AM	*qTFC2.4*	SV_chr2_33658501	SV_chr2_33658501	C	Seq1^c^	Promoter	BLINK	9.58	*Solyc02T000684.1* [27,28]
							CMLM	6.85	
							MLMM	8.35	
							MLM	7.42	
							GLM	7.34	
							FarmCPU	11.60	
	*qTFC6.2*	SNP_chr6_51511965	SNP_chr6_51511965	A	G	Intron	BLINK	6.47	*Solyc06T002703.1*
							CMLM	6.66	
							MLMM	7.73	
							MLM	7.04	
							GLM	7.15	
							FarmCPU	8.01	
MM	*qTFC2.1*	SNP_chr2_26821858	SNP_chr2_26854365	T	C	Promoter	MLM	6.39	*Solyc02T000402.1*
							GLM	6.70	
	*qTFC3*	SNP_chr3_60265480	SNP_chr3_60265480	C	T	Intron	MLM	6.57	*Solyc03T002685.1* [17]
							GLM	6.46	
	*qTFC6.1*	SNP_chr6_49963005	SNP_chr6_49946335	C	T	3’UTR	GLM	7.76	*Solyc06T002476.1* [5,22]
							MLM	7.01	
	*qTFC7*	SNP_chr7_60025223	SNP_chr7_60025223	G	T	Promoter	MLM	6.37	*Solyc07T001849.1*
							GLM	7.16	
	*qTFC9*	SV_chr9_62761281	SV_chr9_62761281	C	Seq2^d^	Promoter	BLINK	6.32	*Solyc09T002159.1*
							FarmCPU	8.55	
	*qTFC10.2*	Indel_chr10_64281583	Indel_chr10_64281583	T	TA	Promoter	CMLM	6.60	*Solyc10T002661.1*
							MLM	7.03	
							GLM	7.05	
SM	*qTFC2.2*	Indel_chr2_28280691	SNP_chr2_28265349	A	G	Intron	GLM	6.52	*Solyc02T000461.1*
			SV_chr2_28264521	Seq3^e^	G	Intron			
	*qTFC2.3*	SNP_chr2_33391697	SNP_chr2_33391697	C	T	Extron, D < ->N	MLM	6.53	*Solyc02T000662.1*
	*qTFC10.1*	SNP_chr10_51854135	SNP_chr10_51758640	C	G	Extron, H < ->D	FarmCPU	6.43	*Solyc10T001673.1*

a AM, the lead variant identified in GWAS results from all six models; MM, the lead variant identified in GWAS results from multiple models (≥ 2); SM, the lead variant identified in GWAS results from a single model.

b Loci or candidate genes, associated with citric acid accumulation found in previous studies have been labeled.

c Seq1: CTTTAATTTTTAATTGATAAATTAATTTCTAATTCATTCATATATCTAATTGATAAATTAATTTTTTGGTTATTAGTGAGCAGGAT.

d Seq2: CGTCTTAGGGATCTTAGCTCGGCCAATATAGGATTTACATGTGCTTATTTTACTTATGAATCGAATTGTCCAGTCACCTCT.

e Seq3: GTATTGTACGCGTACTTGTAACCAAATTGAATTATTGATGAACTTGGTTAAAACCTAAACAAGATGGATTAATATAAATCAATTAATTAACTTCTCAATTCTAAACAACTAAAATAATTAATCTTAATTATGAACTTATTAATTTAAAAAATAAATCTTTTGAGACAATTTTCCGAGATATACTAATTATAGTCAAAATTATATAAAACA.

**Figure 2:**
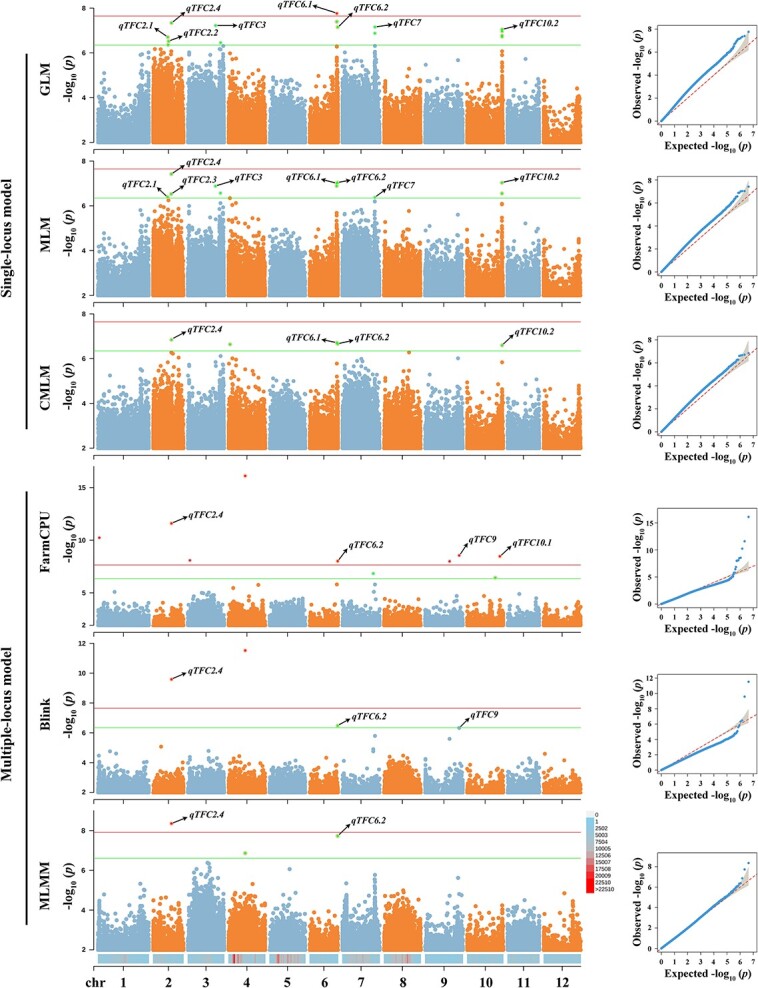
Manhattan and QQ plots with six-mapping models. -log10 (*P*) values from the GWAS results are plotted on the y axis. The red lines indicate genome-wide significant threshold of 7.65, and the green lines indicate suggestive threshold of 6.34. The candidate loci (Table 1) are marked in Manhattan plots. The QQ-plots are at the right of their corresponding Manhattan plots. The distribution of genome-wide variants across 12 chromosomes are shown below the Manhattan plots. Different colors represent the density of variation of markers within 1 Mb windows. The density values are represented with the legend color box on the right.

The GWAS results were analysed through the construction of Manhattan and QQ plots (Fig. 2). QQ plots of multiple-locus models exhibited a line with a strongly deviated tail, which might indicate that these models increased the false positives associated variants. The distributions of CA content were analysed in different genotypes with respect to the lead variants, and seven false positives variants were detected (Fig. S3 and Table S5, see online supplementary material). The findings are in line with those of the QQ plots, as six out of the seven false-positive variants were identified by multiple-locus GWAS. The variants resulting from certain models’ MLM and CMLM (compression MLM) exhibited a close alignment with the 1:1 straight line, indicating that these associated variants could potentially be identified as false negatives. For example, the variant SV_chr9_62761281 was detected from models BLINK (Bayesian information and linkage-disequilibrium iteratively nested keyway) and FarmCPU (fixed and random model circulating probability unification), whereas no signal was observed at this site using the single-locus model (Table 1; Table S2, see online supplementary material). The variants SV_chr2_33658501 and SNP_chr6_51511965 were consistently identified in all GWAS results, demonstrating a remarkably robust association across multiple models. In the MLM-model GWAS, they account for 13.66% and 12.40% of the phenotypic variance explained, respectively. The evidence indicates that quantitative trait loci (QTLs) SV_chr2_33658501 and SNP_chr6_51511965 may serve as the primary loci associated with CA content. Utilizing a multiple-model GWAS approach based on high-density variant markers could yield accurate and effective predictions of CA regulatory genes.

### Candidate genes with variants for tomato citric acid

**Figure 3:**
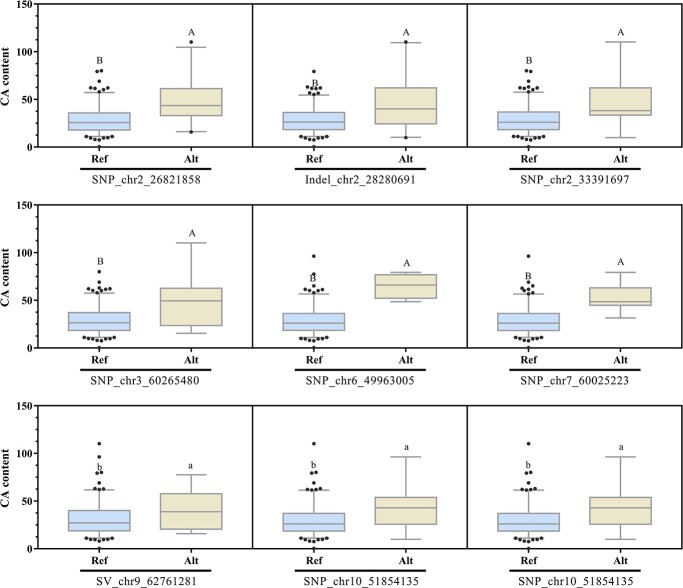
Comparative analyses of CA content in tomato accessions with different lead variants. The distributions as a function of genotypes at the lead variants are analysed, shown as box plots. Different uppercase or lowercase letters represent significant differences at *P* ≤ 0.0001or 0.001 by *t* test, respectively. Ref represents reference allele; Alt represents alternative allele.

**Figure 4:**
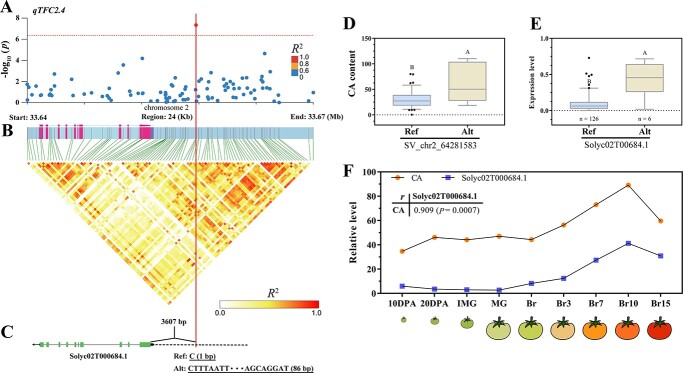
Identification and analyses of the locus *qTFC2.4*. The vertical line denotes the location of structure variation SV_chr2_33658501. (**A**) Detailed plots selected from representative GLM-GWAS result in region 33.64–33.67 Mb (24 kb) on chromosome 2 (x-axis). The dotted lines indicate the significance threshold of *P*-value (4.52 × 10^−7^). (**B**) The heatmap LD depicts the LD block in the 24 kb genomic region corresponding to (**A**). (**C**) The structural model of candidate gene. Green block, exon; Blue block, UTR; Gray line, intron; Black dotted line is the promoter. The black arrow represents the direction of gene transcription. (**D**) Distribution of CA content as a function of genotype at the lead variation. (**E**) Expression of candidate gene *Solyc02T000684.1* in tomato accessions with different genotypes (n: number of accessions). Different uppercase letters represent significant differences at *P* ≤ 0.0001 by *t*-test. Ref represents reference allele; Alt represents alternative allele. (**F**) The correlation between CA content and the transcriptional level of candidate gene *Solyc02T000684.1* during the development of tomato fruits. Pearson’s correlation coefficients *r* and statistically significant correlation value (*P*) are shown.

**Figure 5:**
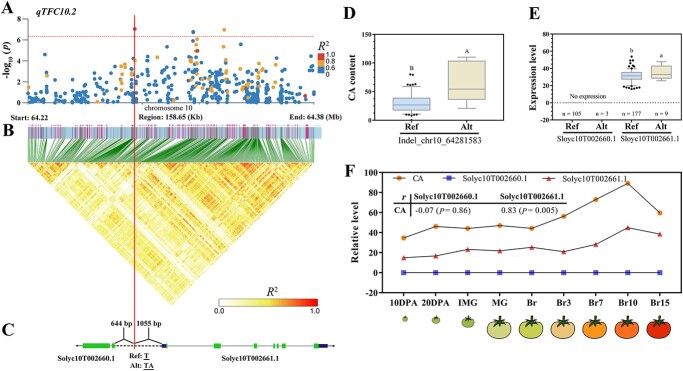
Identification and analyses of the locus *qTFC10.2*. The vertical red line denotes the location of Indel variation on chr10_64281583. (**A**) Detailed plots selected from representative GLM-GWAS result in region 64.22–64.38 Mb (158.65 kb) on chromosome 10 (x-axis). The pairwise *R*^2^ values among all variations are represented with the color legend. The red dotted lines indicate the significance threshold of *P*-value (4.52 × 10^−7^). (**B**) The heatmap LD depicting the LD block in the 158.65 kb genomic region corresponding to (**A**). The *R*^2^ values are marked with the color legend. (**C**) The structural model of candidate genes. Green block, exon; Blue block, UTR; Gray line, intron; Black dotted line, promoter. The black arrows represent the direction of gene transcription. (**D**) Distribution of CA content as a function of genotype at the lead variation. (**E**) Expression of associated genes, *Solyc10T002660.1* and *Solyc10T002661.1* in tomato accessions with different genotypes (n: number of accessions). Different uppercase or lower letters represent significant differences at *P* ≤ 0.0001 or *P* ≤ 0.05 by *t*-test, respectively. Ref represents reference allele; Alt represents alternative allele. (**F**) The correlation between CA content and the transcriptional levels of candidate genes, *Solyc10T002660.1* and *Solyc10T002661.1* during the development of tomato fruits. Pearson’s correlation coefficients *r* and statistically significant correlation value (*P*) are shown.

The CA content varied across different genotypes; therefore, we examined the CA content across diverse genotypes of 21 lead variants (Fig. S3 and Table S5, see online supplementary material). The genotypic analyses of 21 lead variants indicate that all reference genotypes (the genotypes corresponding to the reference genome) were associated with low CA level, suggesting that the reference accession (*Solanum lycopersicum* cv. Heinz) may be lacking in CA content. Seven variants showed no difference in CA content between different genotypes, indicating that they are not effective in distinguishing between high and low CA accessions, and also could not be used to predict candidate genes (Fig. S3, see online supplementary material). The various genotypes of 11 variants (seven SNPs, two Indels, and two SVs) exhibited significant differences in CA content (Fig. 3, Fig. 4D, and Fig. 5D). However, no candidate genes were identified from the three variants based on the variation position and LD block of associated variants (Fig. S4 and Table S5, see online supplementary material). Finally, the 11 variants were ultimately utilized with success in directly predicting causal genes.

The primary objective of GWAS is the precise identification and effective utilization of candidate genes. The results from multiple-model GWAS were synthesized and 11 independent QTLs were obtained, leading to the identification of three distinct types of associated lead variants (Table 1). Among them, the AM type variants (the variants identified in the six-model GWAS) exhibited a remarkably high level of association across multiple models. The QTL *qTFC2.4* (Fig. 4A), classified as an AM type, was identified through the utilization of six statistical models (Table 1). In surrounding DNA region of its lead variant SV_chr2_33658501, no variants exhibiting a stronger LD with lead variant were detected (Fig. 4B). The lead variant, which underwent an 86 bp structural mutation, is located 3607 bp upstream of the gene *Solyc02T000684.1* (Fig. 4C). The tomato accessions with deletion variation exhibited significantly reduced levels of CA content in comparison to those displaying insertion variation (Fig. 4D). Among a total of 123 tomato accessions, the candidate gene *Solyc02T000684.1*, predicted from SV_chr2_33658501, exhibited relatively low expression levels in the accessions of the Ref genotype (Fig. 4E). In addition, the alterations in CA content and the *Solyc02T000684.1* transcription were examined during fruit development (Fig. 4F), showing a significant correlation (*r* = 0.909) between them. The data strongly suggest a significant association between the variant SV_chr2_33658501 and the expression level of the gene *Solyc02T000684.1*; thus, indicating the potential role of AM type variant SV_chr2_33658501 as a causal variation influencing tomato CA content.

The MM-type variants represent the lead variant identified in GWAS results from multiple models (≥2), while the SM-type variants are exclusively identified from a single model (Table 1). Among all nine lead variants (MM and SM types), no causal genes were identified based on the four lead variants (SNP_chr2_26821858, SNP_chr6_49963005, Indel_chr2_28280691, and SNP_chr10_51854135). However, we identified potential variants with high LD values (*R*^2^ = 0.71–0.81; Table S4, see online supplementary material) to these lead variants that could be utilized for predicting candidate genes. The remaining five lead variants were employed for the identification of candidate genes (Table 1). Four of the six MM-type variants are located in the promoter regions of their corresponding candidate genes. The LD region of MM-type QTL *qTFC10.2* was analysed within a span of 158.65 kb (Fig. 5A and B). The lead variant Indel_chr10_64281583 of QTL *qTFC10.2* is positioned within the promoter region of both genes, *Solyc10T002660.1* and *Solyc10T002661.1* (Fig. 5C). The tomato accessions carrying the *Alt* allele of Indel_chr10_64281583 exhibited a significantly high level of CA content (Fig. 5D). Meanwhile, the transcriptional levels of *Solyc10T002661.1* gene in the tomato accessions with this *Alt* allele was found to be low among 286 samples (Fig. 5E). The content of CA showed a significant correlation with the expression of *Solyc10T002661.1* throughout fruit development (*r* = 0.83) (Fig. 5F). The transcript of the *Solyc10T002660.1* gene, however, was not detected in any of the 187 tomato fruit samples (Fig. 5E and F). The data from these results indicate that the variation of Indel_chr10_64281583 may be a causal variant for the differential expression level of the *Solyc10T002661.1* gene, which could potentially be positively associated with CA content.

### Citric acid biosynthesis transcriptionally associated with selected variants

**Figure 6:**
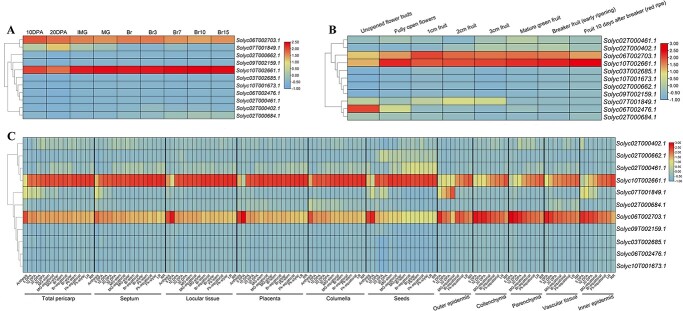
Expression patterns of candidate genes. The expression patterns of candidate genes in tomato fruits from (**A**) *Solanum lycopersicum* MicroTOM (TS-7), (**B**) *S. lycopersicum* Heinz (Reference genome), and (**C**) *S. lycopersicum* M82 (TS-3/228) at different developmental stages were heat mapped. The expression is log_2_-transformed normalized value. Genes are clustered according to their expression patterns.

The gene transcript data from multiple datasets was collected to investigate the expression patterns of candidate genes in order to explore whether these candidate genes exhibit specific expression patterns in fruits (Fig. 6). The dataset unveiled distinct expression patterns, with transcripts of candidate genes detected in at least one tomato fruit tissue. The gene, *Solyc10T001673.1* exhibited a transcripts per million (TPM) value of 0 in both Micro-Tom (Fig. 6A) and Heinz (Fig. 6B), yet its transcripts were detectable in the tomato septum of M82 (Fig. 6C). Additionally, the transcription abundance of *Solyc06T002703.1* and *Solyc10T002661.1* exhibited high levels across the three tomato cultivars in most tissues. The gene *Solyc06T002476.1* also exhibited a high expression level in unopened flower buds of the Heinz cultivar (Fig. 6B). The clustering pattern and heatmap revealed a lack of consistent categorization of gene expression, indicating complex and intricate pattern of gene expressions. For example, the expression of *Solyc02T000461.1* was found to be significantly upregulated during the young fruit stage (1–3 cm fruit) in Heinz cultivar (Fig. 6B), but exhibited high expression levels during the breaker stage in M82 cultivar (Fig. 6C). It was interesting that the gene, *Solyc07T001849.1* exhibited moderate expression throughout the total pericarp (Fig. 6C), but the expression was predominantly observed in the outer epidermis of the pericarp, particularly in the equatorial region of mature fruits. Obviously, the variation in gene expression patterns is clearly attributable to genetic differences.

**Figure 7:**
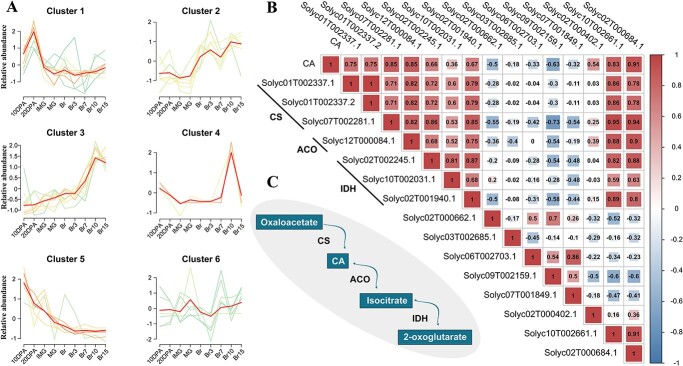
Gene clustering and correlation analyses of the fruit development and ripening. (**A**) The gene cluster identification. The red lines depict the mean trend for each cluster. The average expression trend is highlighted in each cluster. (**B**) The correlation between the variables. The numerical value in each cell denotes the correlation coefficient *r*. (**C**) Pathway module of CA synthesis and metabolism. The probable direction of reversible reactions is indicated by the one-way or two-way arrows.

The CA synthesis and degradation pathway is well understood [3, 8]. The identification of gene clusters was performed by utilizing 40 CA metabolic pathway genes, including malate dehydrogenase, CS, ACO, isocitrate dehydrogenase (IDH), malate synthase, and isocitrate lyase in conjunction with CA-associated genes (Fig. 7A; Tables S6–S8, see online supplementary material). The genes were organized into six clusters, wherein each cluster exhibits consistent expression patterns across various time-points. The gene expression patterns in cluster 3 exhibit a consistent trend that aligns with the changes in CA content throughout the fruit growth cycle. The cluster exclusively contains genes encoding CS, ACO, and IDH enzymes, with their respective positions in CA metabolism (Fig. 7C). We performed correlation analysis to ascertain the association between the seven genes in cluster 3 and CA-associated genes, as well as CA content (Fig. 7B). The analysis revealed an intricate correlation network among the gene clusters.

Interestingly, the expression levels of genes involved in the CA metabolic pathway were found to be positively correlated with CA. Three citrate synthase encoding genes, *CS*, directly control the synthesis of CA. The enzymatic reactions mediated by ACO and IDH in CA metabolism are bidirectional, with a clear preference for CA synthesis rather than degradation. The genes associated with CA exhibited a robust correlation only with the content of CA, but also with the genes involved in the metabolic pathway of CA. The CA content displayed a correlation with the CA metabolic pathway genes, with an *r* value ranging from 0.36 to 0.85. The CA-associated genes (*Solyc02T000684.1*) showed a strong correlation of up to 0.91 *r* value. The transcription levels of *Solyc02T000402.1*, *Solyc02T000684.1*, and *Solyc10T002661.1* presented a positive correlation with the CA metabolic pathway genes and CA content, whereas *Solyc02T000662.1*, *Solyc09T002159.1*, and *Solyc07T001849.1* expression demonstrated a negative correlation with them. These results infer that *Solyc02T402.1*, *Solyc02T000684.1*, and *Solyc10T002661.1* exert a positive regulatory effect on CA accumulation, while the remaining eight CA-associated genes exhibit a negative impact on CA accumulation in tomato fruits.

### Selective sweeps of citric acid associated variants during tomato improvement

A recent study proposed a two-step evolution of fruit mass, involving the domestication of PIM (*Solanum pimpinellifolium*) to CER (*S. lycopersicum var. cerasiforme*) and the subsequent improvement of CER to BIG (*S. lycopersicum*) [29]. Whether the regulatory genes or loci of CA content in tomato fruits have been selected during domestication or improvement, the GWAS analysis enabled us to determine how CA faired or selected during domestication. Thus, the GWAS analysis allowed us to ascertain whether the candidate genes or loci governing tomato CA content were subjected to selection during domestication or improvement, thereby elucidating the impact of domestication on CA content. The subsequent objective was to analyse the selective sweeps of the regions encompassing 11 candidate gene-association variants that underlie the processes of domestication and improvement.

**Figure 8:**
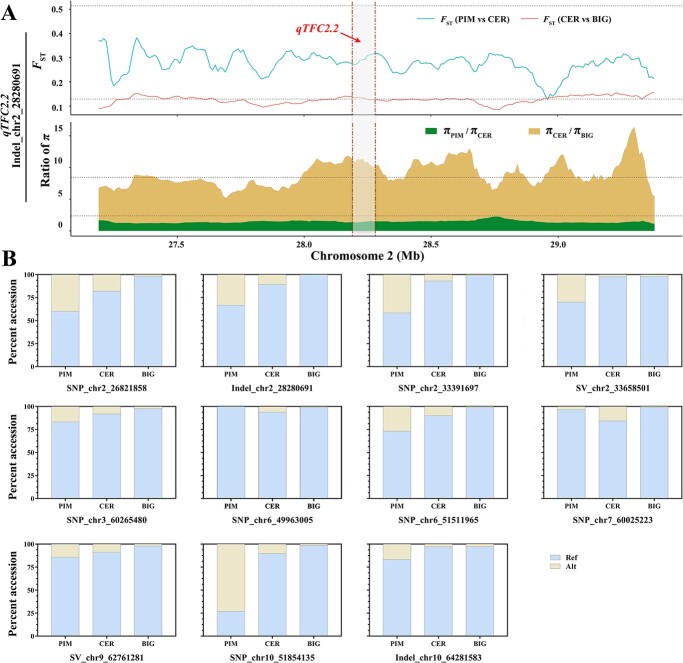
Evolution of CA-associated variations during tomato domestication and improvement. (**A**) Visualization of *F*_ST_ and π ratios for *qTFC2.2* flanking region. The thresholds are labeled with black dotted lines. The two genetic parameters of other 10 loci associated with CA were analysed and shown in Table S9 and S10 (see online supplementary material). (**B**) Distribution of 11 alleles among PIM, CER, and BIG accessions.

The fixation Index (*F*_ST_) (Table S9, see online supplementary material) and nucleotide diversity (π) (Table S10, see online supplementary material) values were compared among PIM, CER, and BIG accessions in the regions of 11 associated loci, and the visualization of two genetic parameters (Fig. 8A; Figs S5 and S6, see online supplementary material). The *F*_ST_ values at QTL *qTFC2.2* did not surpass the thresholds when comparing the genetic differentiation between PIM and CER accessions, whereas there was a high level of differentiation observed between CER and BIG accessions. The π was calculated for the comparison between PIM and CER (π_PIM_/π_CER_), as well as for the comparison between CER and BIG accessions (π_CER_/π_BIG_). The genomic regions of *qTFC2.2* exhibited a significant improvement sweep signal (π_CER_/π_BIG_), while no domestication sweep signal (π_PIM_/π_CER_) was detected. The selective sweeps of an additional 10 regions containing association variants were also investigated (Fig. 8B; Table S10, see online supplementary material). Only *qTFC10.2* exhibited significant domestication sweep signals, as indicated by the significant π_PIM_/π_CER_ values (Table S10, see online supplementary material). The genetic diversity ratios of some variant regions do not exceed the threshold value (8.443) between groups CER and BIG; however, certain variant regions exhibit elevated ratios, such as *qTFC2.1* (6.762), *qTFC2.3* (7.809), and *qTFC7* (5.528).

The detailed distributions of alleles for 11 association variants were analysed in three subpopulations of tomatoes (Fig. 8B). The distribution of alleles exhibited distinct characteristics. The findings clearly indicate a significant decrease in *Alt* distributions from PIM to CER for the majority of alleles across 11 variants. Additionally, there were two variants, SNP_chr6_49963005 and SNP_chr7_60025223, where the *Alt* allele frequency showed improvement during this progression. During the improvement stage, the distribution of *Alt* was observed to exhibit a substantial decrease in most genotypes. Moreover, no discernible differences were found in the *Alt* allele distribution of SV_chr2_33658501 and Indel_chr10_64281583 between CER and BIG, suggesting that these two alleles were not selected during the improvement process. In BIG tomato accessions, the frequency of all *Alt* alleles was extremely low. The data suggest that the high proportion of *Ref* alleles associated with high CA content may be attributed to breeding selection.

### Optimal allelic combinations for citric acid content in tomato fruit

**Figure 9:**
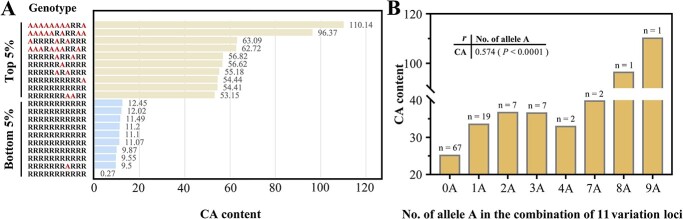
Combinations of the alleles for tomato CA content. (**A**) Genotypes (combinations of 11 candidate gene-associated alleles) with the highest or lowest 5% CA content. The distribution order of the 11 variations position includes SNP_chr2_26821858, Indel_chr2_28280691, SNP_chr2_33391697, SV_chr2_33658501, SNP_chr3_60265480, SNP_chr6_49963005, SNP_chr6_51511965, SNP_chr7_60025223, SV_chr9_62761281, SNP_chr10_51854135, and Indel_chr10_64281583. The CA content of accessions for this genotype is indicated above the column. R represents reference allele; A represents alternative allele. (**B**) Correlations between the number of alternative allele A in the combination of 11 variation loci and CA content. The number of accessions for this genotype is indicated above the column. Pearson’s correlation coefficients *r* and statistically significant correlation value (*P*) are shown.

The diverse tomato accessions exhibited a high level of allelic diversity and displayed distinct allele combinations. The CA-content distributions (Figs 3, 4D, and5D) indicate that the combination of alleles from 11 candidate-gene-associated variations contributes to the CA content in each accession. The complete genotypes of 106 accessions were determined by counting the presence of 11 variants. The tomatoes with the top 5% CA content exhibited a high abundance of alleles A, which were associated with elevated CA concentration (Fig. 9A). Conversely, the tomatoes with the bottom 5% CA content displayed a significant prevalence of alleles R, linked to low CA concentration (Fig. 9A). The genotype AAAAAAAARRA, which possesses nine alleles with high CA content (allele A), exhibited the highest CA content among all accessions in the GWAS population. The genotype RRRRRRARRRR, despite possessing one A allele, was classified as the low-CA tomato due to incongruences. With the increase in the total number of genotype A at the 11 variation loci combinations, the CA content was maintained at a relatively high level (Fig. 9B; Table S11, see online supplementary material).

The presence of fewer individuals in certain genotypes resulted in inadequate statistical power for high proportions, yet there existed a strong correlation (*r* = 0.574, *P* < 0.0001) between the allele A count and CA content. Based on the comparison of tomato CA content among different genotypes with variations (Fig. 9), it can be inferred that the putative genotype for high CA content is AAAAAAAAAAA (11A) and low CA accessions should possess RRRRRRRRRRR (11R) genotype. In fact, the genotype 11A was not present in our population, and only a small number of individuals possessed more than seven alleles of A. The Type R alleles of associated variants exhibited the highest frequency of distribution (Fig. 9B), resulting in a substantial proportion of individuals with the 11R-genotype. The prevalence of low CA-content tomato accessions in this population could potentially be attributed to this phenomenon (Fig. 1A).

## Discussion

The focus of breeders has been on enhancing the commercial traits of tomatoes, including but not limited to increased yield, resistance to pests and diseases, longer shelf life, and fruit color [30–32]. However, the weight of tomato fruit exhibits a negative correlation with major flavor substances, including acids (such as CA and malic acid), sugars (fructose and glucose), as well as various volatile compounds [24, 29]. Consequently, modern commercial tomato breeding prioritizes high yield at the expense of flavor [22]. Presently, breeders are directing their attention towards the optimization of taste and nutritional value in fruits. The advancement of genetic engineering of major crops to improve commercial traits has been bolstered by technological innovations, albeit impeded by the limited genetic variability inherent in cultivated crops. The assessment of the genetic underpinnings of flavor profiles constitutes an indispensable prerequisite for the advancement of variety development. CA not only serves as a mediator in a series of metabolic pathways, but also plays a crucial role as an essential flavor acid in fruits [3, 33]. The accumulation of CA is determined by a complex regulatory network [3, 7, 8, 20]. The genetic enhancement of key crops for improved traits has been facilitated by technological advancements, yet impeded by the limited genetic diversity within cultivated crops. GWAS has become a classical genetic approach utilized to identify associated loci and causal genes linked to breeding traits in crops [5, 22, 26]. In the present study, we conducted a genome-wide association study to investigate the loci associated with fruit CA content based on SNP, Indel, and SV, as well as predicting causal genes based on the identified variants.

### Benefit of multi-model GWAS utilizing genome-wide variations

Previous studies using GWAS have explored the association between natural variation and CA metabolism in tomatoes [4, 24]. Unfortunately, the yield of significant variant sites associated with CA content was limited to zero or only one [24, 25]. The outcomes of GWAS analyses are influenced by various factors, including the association model, population structure, phenotype, and environmental influences [23]. The detection of variations associated with CA in tomato fruits content was comprehensively conducted through genome-wide variant-based GWAS in this study. The primary concern in model selection lies in the management of false positives or false negatives [34]. This study employed six multiple/single-locus models to establish associations between variants and phenotypes of varying complexity, thereby ensuring comprehensive analyses. The statistical power of GWAS is further impeded by genomic variants. Findings from earlier studies have demonstrated that SVs account for the largest proportion of the overall heritability explained by SNPs, Indels, as well as SVs [26], while Indels contribute to explaining certain phenotypic variations [35].

Two SVs and two Indel lead variants associated with CA content were detected in the present study. As a subset of high-density variations, consisting of 4 353 430 variants (4 063 982 SNPs Indels and 55 117 SVs), was sufficiently comprehensive to enable the detection of GWAS signals using multiple mapping models in our study. We identified 11 promising candidate genes with associated causal variants, which can be categorized into three types: AM, MM, and SM (Table 1). The presence of AM-type loci was observed in all models, demonstrating a consistent signal for identifying candidate genes. The presence of a *TFM6* locus associated with malic acid has consistently been observed across various GWAS models within a single study (25), establishing this QTL as a prominent genetic factor influencing malic acid accumulation in tomato [4, 29, 36]. Also, MM-type SNPs were obtained from the GWAS results with several models, indicating a moderate level of confidence. Previous studies have demonstrated that QTL *qTFC6.1* is a genetic locus associated with the content of CA [5, 22]. Moreover, another two models, ECMLM (Enriched CMLM) and SUPER (Settlement of MLM Under Progressively Exclusive Relationship) were carried out to test lead variants (Fig. S7, see online supplementary material). The repeated detection of several loci, such as *qTFC2.4*, *qTFC6.1*, and *qTFC10.2*, is evident. Our data suggest that multi-model GWAS utilizing variations across the entire genome is highly advantageous in capturing additional heritability for CA content in tomato. In order to comprehensively decipher the genetic basis of complex quantitative traits, it is imperative to place greater emphasis on the variant types of Indels and SVs, rather than exclusively focusing on SNPs.

### High confidence in identification of citric acid regulatory genes

The identification of candidate genes is a primary objective of GWAS, by which candidate genes with loci serving as central regulators for CA metabolism are expected to be identified [37]. The study has convincingly shown that genome-wide variation possesses a capability to identify causal variants in complex traits of CA accumulation. The AM-type loci *qTFC2.4* suggests *Solyc02T000684.1* as a potential candidate gene, with its causal variant SV_chr2_33658501 occasioning an SV variation in its promoter region that triggers differential transcriptional expression (Fig. 4). The gene *Solyc02T000684.1* encodes a phospholipase D protein, and the functional significance of its gene family member phospholipase C in living organisms has long been recognized, with its crucial role in cell signal transduction being widely acknowledged [27, 28]. The phospholipase C signaling pathway transmitted by the Al^3+^ signal may be involved in the stimulation of citrate transport by activating transcription and anion channels in the plasma membrane [27]. Phospholipase C-mediated pathways modulate CAMTA2 and WRKY46 to regulate the Al-inducible *Al*-*ACTIVATED MALATE TRANSPORTER* (*ALMT*) expression [28]. Meanwhile, the overexpression of *ALMT* leads to a substantial elevation in the levels of CA [36]. The tomato carrying the high allele in SV_chr2_3365850 exhibited relatively elevated expression of *Solyc02T000684.1* and higher CA content (Fig. 4D and E). The CA content and the transcription level of *Solyc02T000684.1* exhibited an extremely significant correlation throughout the process of fruit development (Fig. 4F). Additionally, the expression of *Solyc02T000684.1* showed a strong positive correlation with the content of CA and the transcription of genes encoding enzymes involved in the CA metabolic pathway, namely CS, ACO, and IDH (Fig. 7) [3, 8]. The data strongly suggests a positive correlation between the phospholipase protein and CA content. The detection of three ubiquitylation pathway proteins in GWAS, namely ubiquitin family member (*Solyc02T000662.1*), F-box protein (*Solyc10T001673.1*), and RING/U-box superfamily protein (*Solyc09T002159.1*), suggests the involvement of ubiquitination degradation process in regulating the CA metabolic process in tomato. The involvement of ubiquitin-protein in the dynamic modulation of citric acid accumulation has been demonstrated in citrus fruits [38]. The transcription factor plays a crucial role in the accumulation of CA in citrus [39]. This study identified a *basic Helix–Loop–Helix* (bHLH) DNA-binding transcription factor, *Solyc03T002685.1*, associating with tomato fruit CA content. The bHLH transcription factor has been demonstrated to regulate the expression of *ACO* genes, thereby controlling the accumulation of CA [17]. The weight of tomato fruit was significantly and inversely correlated with CA content than other flavor metabolites, leading to a decline in the sensory perception of tomato fruit taste [24, 29]. Thus, the replacement of low CA alleles is expected to significantly enhance consumer preferences [22]. The novel genes from this study may offer valuable insights into the genetic enhancement of CA accumulation in tomato.

### Decreased citric acid level in fruits attributable to tomato improvement stage

The CA content exhibited diverse variations consistent with previous studies [5]. It is intriguing to speculate on the impact of selection during domestication on CA content in tomato breeding. In our study, no discernible phenotypic difference was observed in the CA content between PIM and CER accessions (domestication), but the BIG accession showed a lower CA content compared to PIM (improvement) or CER accession (Fig. 1E). Based on the genetic parameters of population differentiation, a significant sweep signal was identified, and the genomic regions of 10 other variations exhibited high π_CER_/π_BIG_ ratio values during the improvement stage (Fig. 8A; Table S10, see online supplementary material). In addition, the distribution of *Alt* was observed to exhibit a substantial decrease across most genotypes in this breeding stage (Fig. 8B). The above observation has been reported in other studies, demonstrating a significant difference in CA content between CER and BIG accessions [4, 22].

During domestication, a significant decrease in *Alt* distributions was observed. However, the *Alt* allele frequency of two variants exhibited an increase during this progression, which could, at least partly, explain the absence of any discernible phenotypic difference. The comparison of PIM and CER in another study revealed a significant difference in CA content [22]. We speculate that the contribution to phenotype differences can be attributed to both environmental and genotypic variations. The regulation of fruit secondary metabolism is significantly influenced by environmental factors [40]. Genetic background determines the CA concentrations of PIM, CER, and BIG tomato fruits, whereas environmental factors may account for prominent quantitative changes to CA levels. Temperature, nutrient availability, water supply, light exposure, biotic and abiotic stresses have been demonstrated to impact the biosynthesis of secondary metabolites in fruits [41, 42]. In sum, these findings are suggestive of an improvement event that CA content in tomato is more influenced by improvement sweeps with minimal influence from domestication sweeps.

### Efficient tools to improve citric acid in tomato with multiple loci

The techniques of genotyping, marker-assisted selection, and genomic selection can be employed for expedited breeding [37]. The application of marker-assisted selection has proven to be successful in the field of crop breeding. The utilization of whole-genome high-throughput genotyping platforms, array-based genotyping, and PCR-based markers is indispensable in marker-assisted breeding [43]. The primary challenge in genotyping, however, lies in its exorbitant cost. Next-generation sequencing technologies continue to be prohibitively expensive, while PCR-based markers remain a labor-intensive and time-consuming method of genotyping. Moreover, the application of molecular marker-assisted breeding in crop improvement is limited by the presence of complex traits controlled by multiple small-effect loci.

Array-based genotyping platforms offer a flexible solution for customized probe detection, making them an essential tool in this regard. Currently, high-density SNP genotyping arrays have been developed and utilized extensively in marker-assisted breeding of crops [43, 44]. The presence of numerous variants with low effects significantly impacts the breeding for complex traits, such as CA [4, 24, 26, 45, 46]. It is crucial to identify variations associated with CA at the whole genome level in order to facilitate targeted breeding for CA improvement. If the variants are located within annotated genes or promoter regions, the candidate genes selected for CA genotyping analysis will be those with high-confidence annotation [43]. Additionally, the number and percentage of SNP variants located within gene intervals and associated genes are important indicators for evaluating the quality of SNP chips [37, 47]. In the present study, we identified 11 candidate gene-association variants related to CA content, which can be utilized for the development of precise function-based markers in the selection and breeding of CA-rich tomato varieties. Thus, gene function-based markers for CA content are essential for marker-assisted breeding, and our study provides crucial variant information for the development of breeding tools such as SNP arrays.

## Materials and methods

### Tomato genetic resources and genomic data

The GWAS population was derived from a classically natural population, which outlines the historical processes of tomato domestication and improvement [29]. In our study, this natural population, including 18 *S. pimpinellifolium* (PIM), 91 *S. lycopersicum var. cerasiforme* (CER), and 131 *S. lycopersicum* (BIG), was utilized for the identification of genome-wide variants and for conducting the GWAS. The genomic information of 240 tomato accessions can be accessed via the National Center for Biotechnology Information Sequence Reads Archive (SRA), with the accession number: SRP045767 [29].

### Phenotypic assessment

The materials were cultivated in the greenhouse of Huazhong Agricultural University, Wuhan, China, with a temperature of 25 ± 2°C, relative humidity of 70%, and a photoperiod of 16 hours light and 8 hours dark during the growth period. Each sample consisted of a minimum of three plants, each bearing at least three fully matured fruits. The samples were lyophilized with liquid nitrogen and subsequently stored at −80°C for the quantification of CA levels. The CA content was quantified using the method previously described [36]. Reliable phenotypic data from the 240 accessions were obtained for constructing association mapping panels.

### Detection of genetic variants

To perform a variant calling on the tomato accessions using short reads sequencing, all paired-end sequence reads were first cleaned using a FASTQ preprocessor [48]. The high-quality cleaned reads were then aligned to the tomato Graph pangenome TGG1.1 (SL5.0 serving as the backbone, https://solgenomics.net) [49] with *giraffe* function implemented in variation graph toolkit (vg) [50]. The reads files aligned to graph for SNP calling were filtered according to the parameters: -r 0.90, −fu, −m 1, −q 15, and -D 999. The alignment reads with low quality, defined as mapping qualities and positions with base quality less than 5, were subsequently excluded using the ‘*vg pack*’ tool with the parameter ‘-Q 5’. The variant calling for each sample was executed utilizing vg call.

All individual variants were merged and normalized using the BCFtools software [51]. A comprehensive genomic variant dataset was acquired, encompassing SNPs, Indels (1–50 bp) as well as SVs (>50 bp). The high-quality variants were subjected to further filtering based on the following criteria: a MAF of ≥5% for indels and SNPs, or ≥2.5% for SVs, and a missing rate per site of ≤50%. Finally, a subset of 4 353 430 variants across the 240 tomato accessions was selected and utilized for further analyses. PLINK was used to calculate the heterozygosity for each individual accession [52]. The plot depicting the distribution and density of variants was generated utilizing the R package *CMplot* [53].

### Variant-based heritability estimation

The GREML method [54] was used to estimate the proportion of variance in a phenotype explained by all variants with GCTA tool [55].

### Genome-wide association study

Based on detected genome-wide variants, the correlation between variant loci and CA trait were assessed with six association mapping models, including MLM [56], GLM [57], CMLM [58], MLMM [59], FarmCPU [60], and BLINK models [61]. Models GLM, MLM, and CMLM are single-locus models, while models MLMM, FarmCPU, and BLINk are multi-locus models. Within the category of single-locus models, CMLM is superior to MLM, and MLM is superior to GLM. Within the category of multiple-loci models, BLINK is superior to FarmCPU, while FarmCPU is superior to MLMM [62].

GWAS was performed using the *R* package *GAPIT* (version 3), employing the association mapping models as described above [62]. For all six models, the first three principal components were utilized to account for population structure correction. The genetic relatedness was determined by the VanRaden kinship matrix [63]. The thresholds for genome-wide significance were established at suggestive (1/n, where n represents the effective number of independent variants, 4.52 × 10^−7^) and significant (0.05/n, 2.26 × 10^−8^) *P*-values to identify variant loci with statistically significant associations. The effective number of independent variants determined using Genetic type 1 Error Calculator software (http://grass.cgs.hku.hk/gec/download.php) [64]. The R package *CMplot* was utilized for the visualization of GWAS results, including Manhattan plots and QQ plots [53].

### Identification of causal variants and linked candidate genes

Comparative analyses of CA content in tomato accessions with different lead variants, suggestive variants (*P* < 4.52 × 10^−7^), were used to exclude inaccurate false positive variants. The verified variants were recognized as associated variants with CA, and utilized to ascertain potential candidate genes that regulate the accumulation of CA. The associated variants were classified into independent QTLs based on LD analysis using PLINK software [52], and the correlation coefficient (*R*^2^) was calculated to determine pairwise LD decay. Variants exhibiting high LD levels with the peak variant (*R*^2^ = 0.6–1) were deemed to be located within an LD region. Visualizing LD and haplotype blocks were constructed using the LDBlockShow software [65].

The physical locations and mutation types of associated variants were determined using the snpEff tool [66] based on the tomato reference genome (version SL5.0), and candidate genes were annotated according to their corresponding annotation information (http://solomics.agis.org.cn/tomato/). If there were potential causative relationships between the significant variants and the candidate genes, these variants were considered as causal variants, and their corresponding genes identified as candidate genes. If not, the prediction of candidate genes was based on the linkage disequilibrium region where the peak variant is located. The peak variants or the variants in high LD with the peak variant are directly co-localized with annotated genes (resulting in amino acid mutations) or situated within the promoter regions (2 Kb upstream of the gene). In this case, these variants and corresponding genes were considered to have a causal relationship with CA.

### Expression profiles of candidate genes

Data from RNA-seq were filtered by FASTQ [48], and the transcript abundances were quantified (https://solgenomics.net/ftp/genomes/TGG/cds/) using the Kallisto software (v.0.46.2) [67]. The TPM values obtained from the output were utilized for quantifying gene expression levels. The expression of genes in various organs and fruits at different stages was crosschecked in three genomic backgrounds of tomato, namely *S. lycopersicum* Micro-Tom [68], *S. lycopersicum* Heinz 1706 [69], and *S. lycopersicum* M82 [70]. The RNA-Seq data from 526 samples (483 samples from M82, 27 samples from Micro-Tom, and 16 samples from Heinz) were quantified.

The transcriptional levels of candidate genes in tomato fruits were analysed in tomato accessions with diverse genotypes. The RNA-seq data from fruit pericarp tissue with diverse genotypes was obtained from the SRA PRJNA396272 database [4]. The correlation between the CA content and the expression level of candidate genes during tomato fruit development was also examined. The CA content values for nine developmental stages of tomato fruits (*S. lycopersicum* Micro-Tom) were obtained from previous study [68]. The RNA-seq datasets for gene expression profiles can be accessed at the Genome Sequence Archive at the Big Data Center with the accession number: CRA001723 (http://bigd.big.ac.cn/gsa). The gene expression patterns were visualized using the TBtools software with Heatmap plugin [71].

### Gene cluster identification and correlation analysis

The metabolic pathway genes of CA were accessed from the Kyoto encyclopedia of genes and genomes (KEGG) database (https://www.kegg.jp/pathway/map00020). The TPM values of the genes encoding six key enzymes, namely malate dehydrogenase, CS, ACO, IDH, malate synthase, and isocitrate lyase, were utilized for gene cluster identification in conjunction with CA-associated genes. The RNA-seq data of 27 samples from nine developmental stages of tomato fruit were downloaded from the Big Data Center (http://big.big.ac.cn/gsa) using the accession number: CRA001723. Normalized TPM values of genes were clustered into six groups using Hiplot (https://hiplot.cn) with default parameters. The correlation between the expression of cluster 3 and CA-associated genes, as well as the content of CA, was analysed based on the *r* value of Pearson’s correlation coefficient [72].

### Detection of domestication and improvement sweeps

The π was employed as a metric to quantify the extent of genetic variation within our GWAS population [4, 29]. The *F*_ST_ was utilized to validate regions of molecular diversity that exhibit high levels of differentiation [36]. The selective sweeps signals related to CA content during tomato evolution were identified through the application of π and *F*_ST_ analyses at two crucial stages, namely domestication and improvement [29]. The parameters were calculated using the VCFtools package [73] with a 100 kb sliding window and a step size of 10 kb for genome-wide scanning in PIM, CER, and BIG. The windows exhibiting the top 5% of genetic diversity ratios between PIM and CER (π_PIM_/π_CER_) as well as between CER and BIG (π_CER_/π_BIG_) (2.367 and 8.443 for domestication and improvement, respectively), along with population-differentiation ratios, *F*_ST_ (PIM vs CER) and *F*_ST_ (CER vs BIG) (0.514 and 0.129 for domestication and improvement, respectively) were identified as regions undergoing selective sweeps.

## Acknowledgements

This work was supported by grants from the National Key Research & Development Plan (2022YFD1200502; 2021YFD1200201); National Natural Science Foundation of China (32372696; 31991182); Wuhan Biological Breeding Major Project (2022021302024852); Key Project of Hubei Hongshan Laboratory (2021hszd007); HZAU-AGIS Cooperation Fund (SZYJY2023022); Funds for High Quality Development of Hubei Seed Industry (HBZY2023B004-1; HBZY2023B004-6); Hubei Agriculture Research System (2023HBSTX4-06); Hubei Key Research & Development Plan (2022BBA0066; 2022BBA0062).

## Author contributions

W.G. and Y.Z. conceived and designed the experiments. W.G., L.Y., and F.Y. analysed the data and carried on the analyses. J.T. and X.Z. performed the data collection. J.K.A., F.L., P.G., F.W., Y.Y., and Y.Z. conducted the literature review and edited the manuscript. Y.Z. supervised the project and revised the manuscript.

## Data availability

All the other supporting data are included in the article or the supplementary files.

## Conflict of interest statement

The authors declare no competing interests.

## Supplementary data

Supplementary data is available at *Horticulture Research* online.

## Supplementary Material

Web_Material_uhae070
