# Supplementary material for: Genome-wide variants and optimal allelic combinations for citric acid in tomato
Source: Hortic Res. 2024 Mar 18;11(5):uhae070. doi: 10.1093/hr/uhae070 (PMC11079488; doi:10.1093/hr/uhae070)
Supplement: Web_Material_uhae070 [file web_material_uhae070.zip › Supplementary materials.pdf]

Supplementary materials

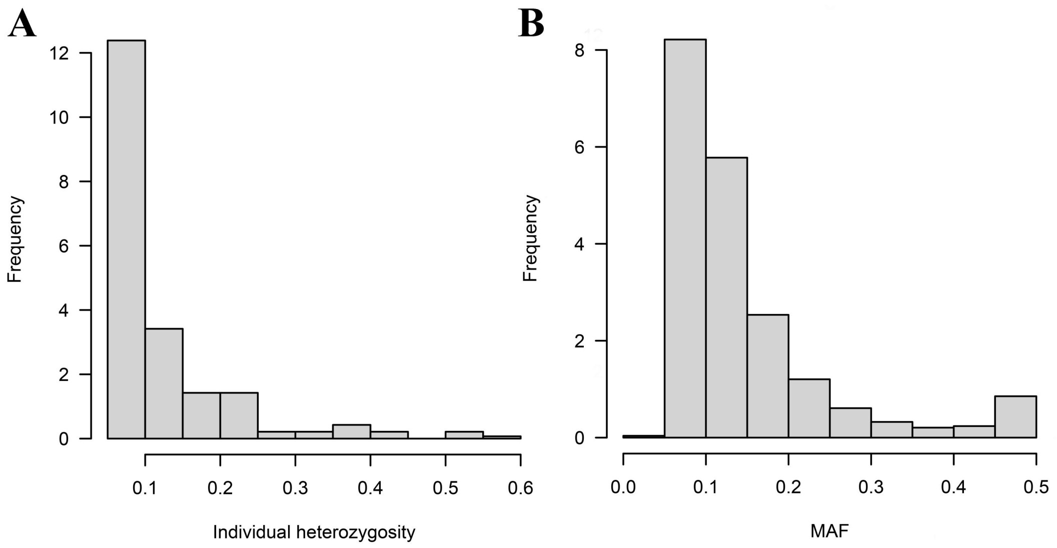

Supplementary Fig. S1 The frequency of individual heterozygosity (A) and the distribution of per-variant MAF (B).

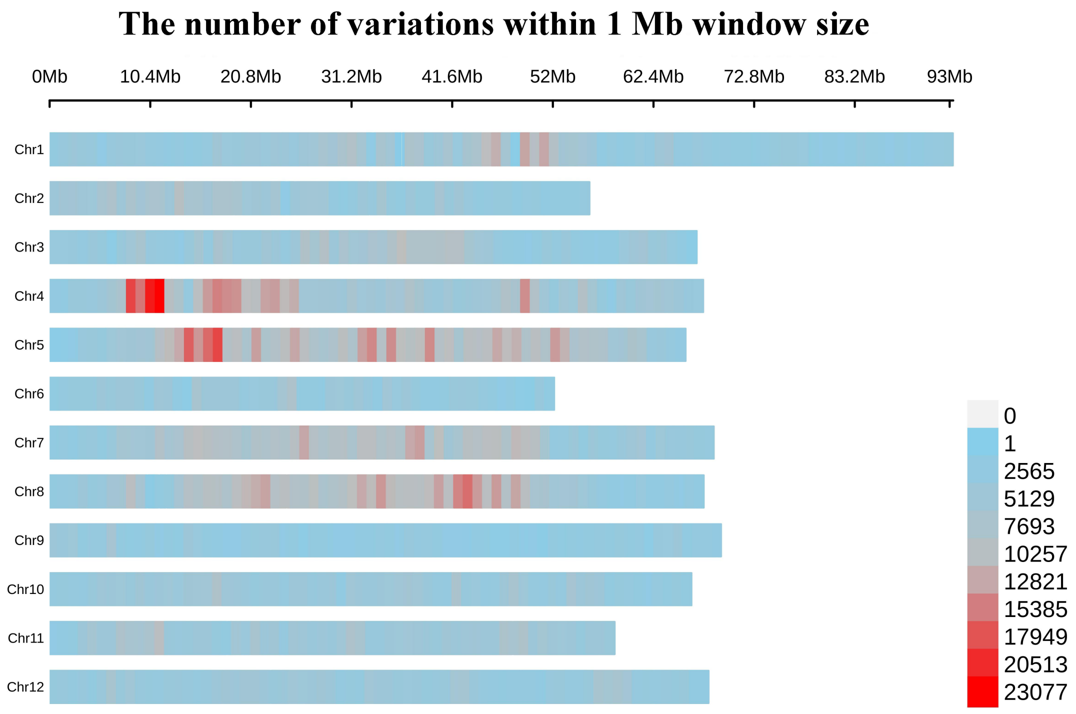

Supplementary Fig. S2 The distribution of genome-wide variations across 12 chromosomes. Different colors represent the density of variant markers within 1 Mb windows. The density values are represented with the legend color box on the right.

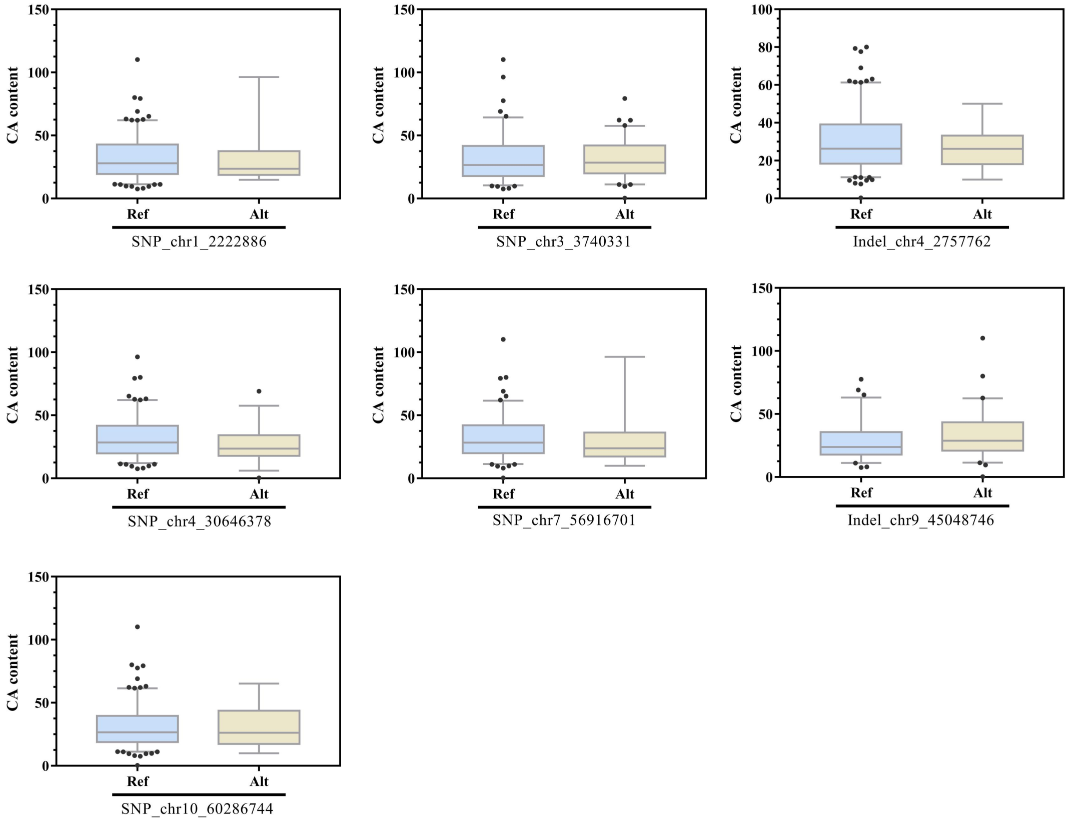

Supplementary Fig. S3 Comparative analyses of tomato CA content with seven different lead variants (no deference). The distributions as a function of genotypes at the lead variant are analyzed, shown as box plots. Ref represents reference allele; Alt represents alternative allele.

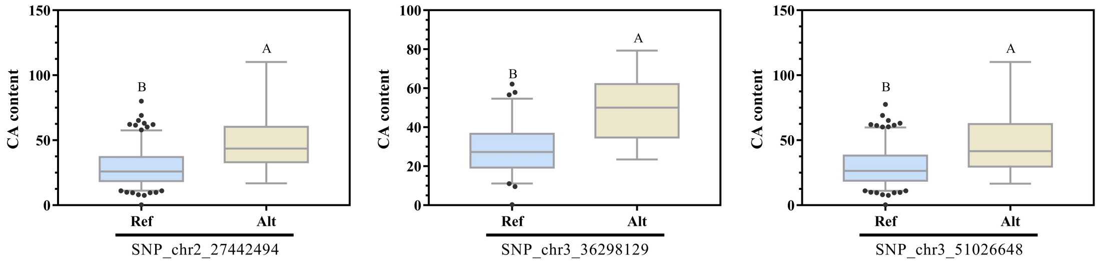

Supplementary Fig. S4 Comparative analyses of tomato CA content with three different lead variants. The distributions as a function of genotypes at the lead variant are analyzed, shown as box plots. Different uppercase letters represent significant differences at $P \leqslant 0.0001$ by t test, respectively. Ref represents reference allele; Alt represents alternative allele.

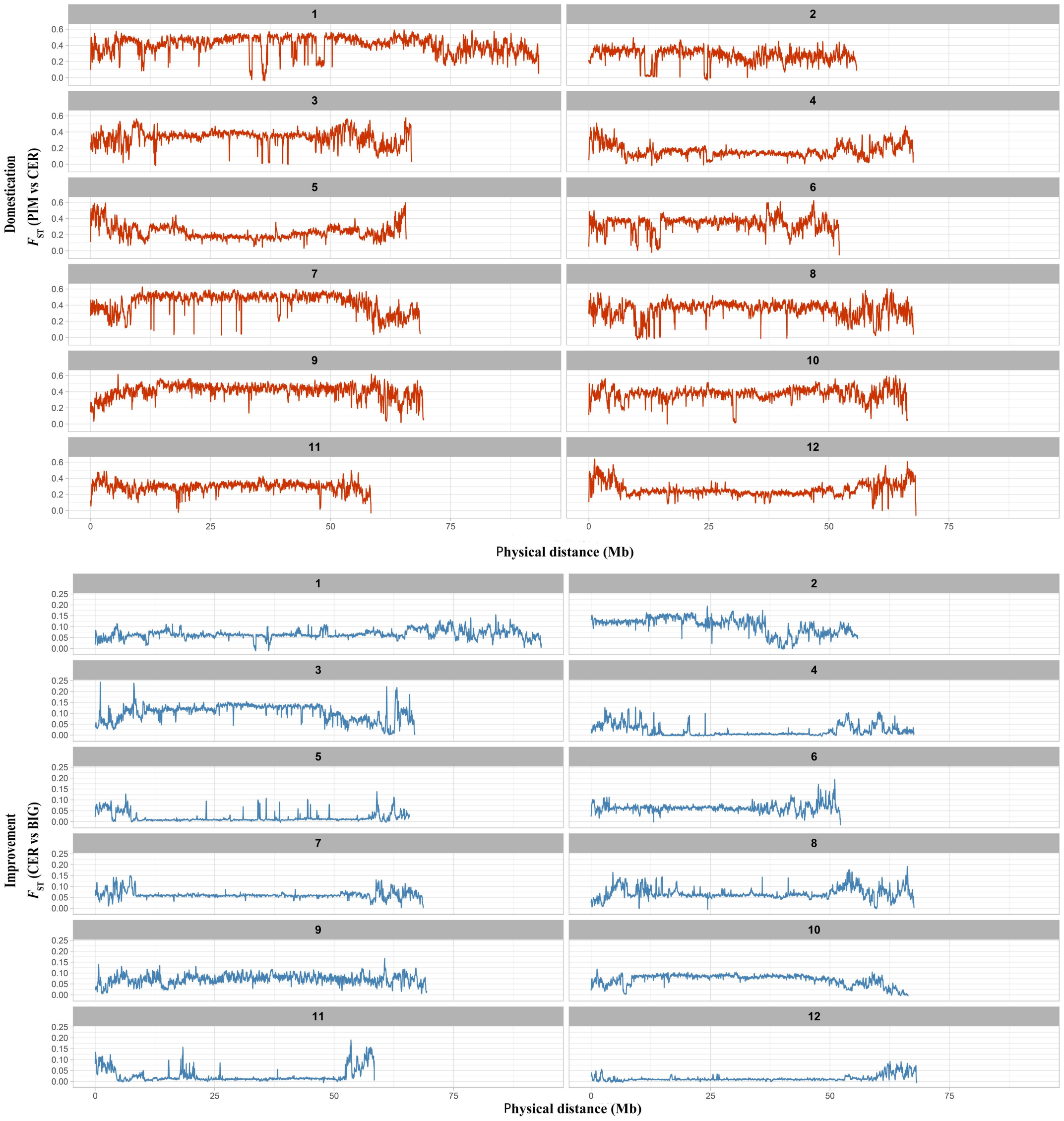

Supplementary Fig. S5 F_{ST} values for all variant sites during domestication and improvement.

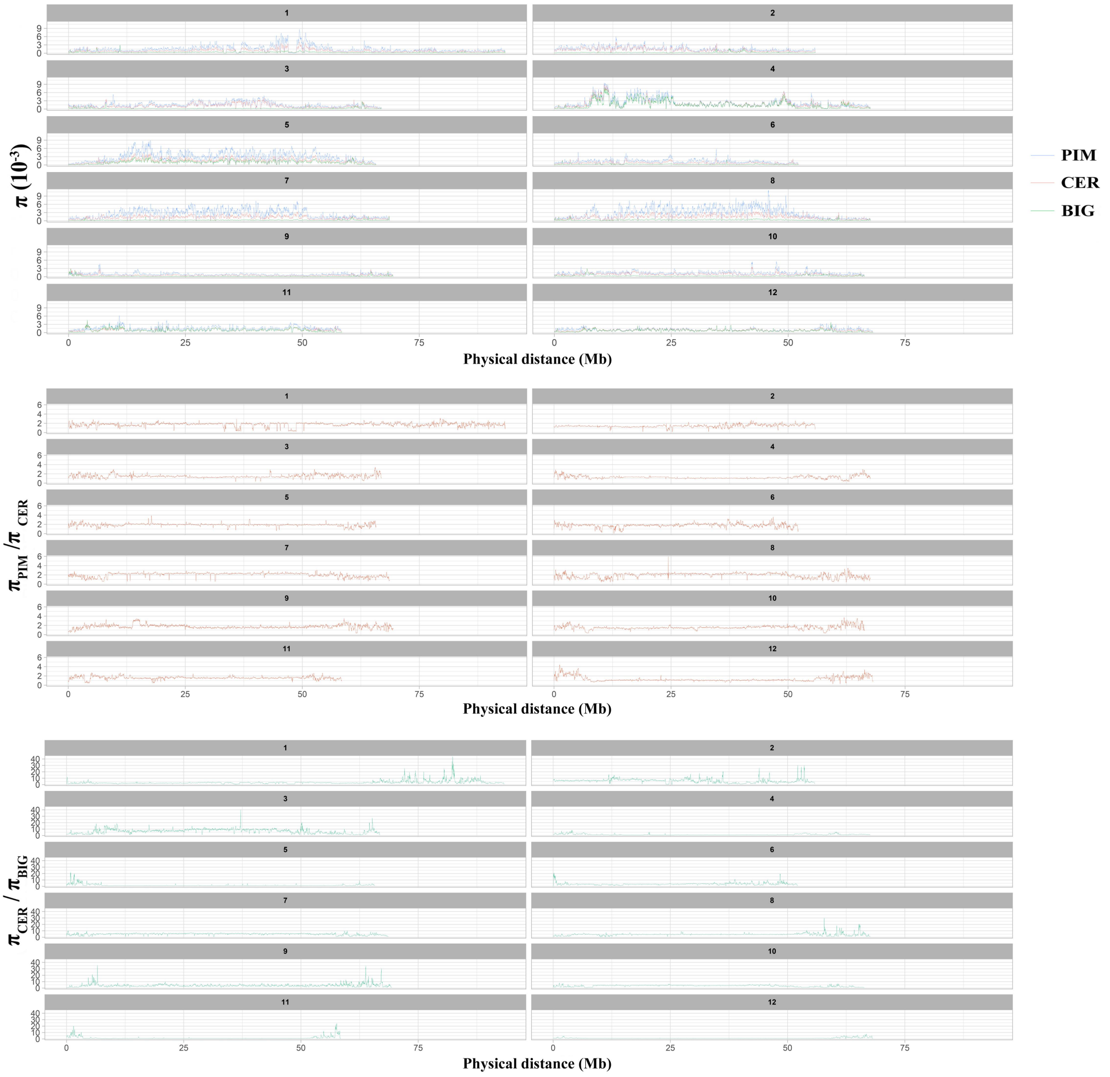

Supplementary Fig. S6 π and π ratios values for all variant sites during domestication and improvement.

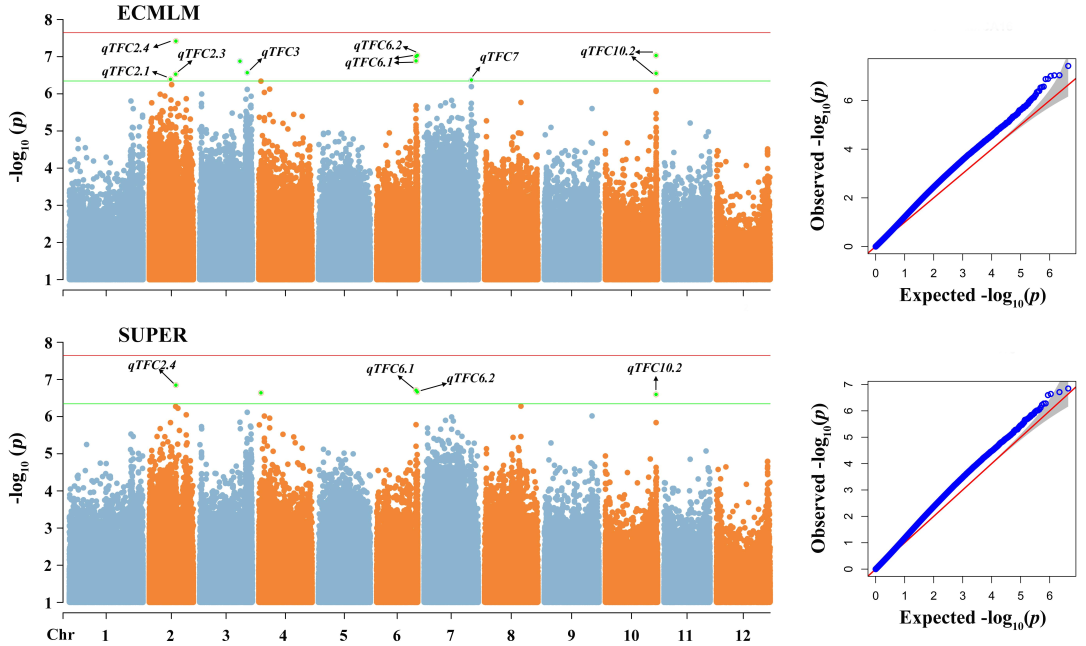

Supplementary Fig. S7 Manhattan plots with ECMLM and SUPER models. ECMLM and SUPER models are carried out using GAPIT software (http://zzlab.net/GAPIT). -log10 (*p*) values from the GWAS results are plotted on the y axis. The blue lines indicate genome-wide significant threshold 7.65, and the orange lines indicate suggestive threshold 6.34. The QQ-plots are at the right of their corresponding Manhattan plots.

Supplementary Table S1 Details of the variants number utilized for GWAS analyses.

Supplementary Table S2 Comparison of the number of associated variants, loci, and candidate genes identified by multiple GWAS methods.

Supplementary Table S3 The summary of all significant variants identified by genome-wide association analysis. For each model type, model, chromosome, position (bp), variation, *P*. value, and minor allele frequency (MAF) are provided.

Supplementary Table S4 Details of locs associated with tomato CA. For each locus, lead/casual variant (variant-type_chromosome_position), reference allele (Ref), alternative allele (Alt), position (bp), model, -log10 (*p*), and candidate genes are provided., and so on are provided.

Supplementary Table S5 Details of the variants without candidate genes. For each associated variant (variant-type_chromosome_position), chromosome, reference allele (Ref), alternative allele (Alt), model, -log10 (*p*), and models are provided are provided.

Supplementary Table S6 F_{ST} values of the flanking regions of variants associated with putative genes during domestication and improvement.

Supplementary Table S7 π values of variants associated with putative genes during domestication and improvement.

Supplementary Table S8 The genotypes of candidate genes-related variants in 207 tomato accessions. R represents homozygous reference allele. A represents

homozygous alternative allele. H represents heterozygous allele. 0 represents the deletion of allele.

Supplementary Table S9 The TPM values utilized in Fig. 6.

Supplementary Table S10 Summary of co-expression gene clusters.

Supplementary Table S11 Description of the metabolic pathway genes.
